# Supplementary material for: Chitosan-Urea Nanocomposite for Improved Fertilizer Applications: The Effect on the Soil Enzymatic Activities and Microflora Dynamics in N Cycle of Potatoes (Solanum tuberosum L.)
Source: Polymers (Basel). 2021 Aug 27;13(17):2887. doi: 10.3390/polym13172887 (PMC8433729; doi:10.3390/polym13172887)
Supplement: Supplementary file 1 [file polymers-13-02887-s001.zip › polymers-1349020-supplementary.pdf]

Supplementary Table 1. Effect of application of different concentrations of nano-chitosan-urea composite and conventional urea fertilizer on the soil chemical properties

N-level	DAS	pH			EC (dS m^{-1})			OC (%)		
		CU	NCUC	CS	NCUC	CS	CS	CU	NCUC	CS
**0**	0	7.03	7.04	7.03	0.30	0.27	0.30	1.57	1.57	1.57
	20	7.13	7.12	7.13	0.56	0.75	0.56	1.52	1.53	1.52
	40	7.36	7.44	7.36	0.45	0.78	0.45	1.34	1.38	1.34
	60	7.43	7.42	7.43	0.56	0.74	0.56	1.22	1.31	1.22
	90	7.59	7.52	7.58	0.45	0.65	0.45	1.23	1.31	1.23
**50**	0	7.02	7.04	7.04	0.24	0.23	0.26	1.53	1.53	1.58
	20	6.85	6.97	6.8	0.81	1.31	0.84	1.39	1.35	1.46
	40	7.11	7.47	7.12	0.73	0.85	0.84	1.28	1.06	1.10
	60	7.45	7.44	7.44	0.73	0.82	0.74	1.02	0.86	0.88
	90	7.83	7.74	7.81	0.65	0.63	0.63	0.68	0.53	0.66
**75**	0	7.04	7.04	7.03	0.23	0.23	0.24	1.55	1.55	1.54
	20	6.92	7.14	6.78	0.84	1.52	0.84	0.83	0.76	0.87
	40	7.49	7.48	7.27	1.64	0.72	0.87	0.83	0.57	0.52
	60	7.67	7.5	7.65	1.32	0.84	0.75	0.53	0.53	0.54
	90	7.87	7.74	7.81	0.67	0.74	0.64	0.34	0.33	0.55
**100**	0	7.03	7.03	7.03	0.22	0.24	0.25	1.53	1.55	1.54
	20	7.22	7.29	7.03	1.30	1.31	0.46	0.86	0.73	0.75
	40	7.56	7.34	7.29	1.47	0.95	0.57	0.77	0.55	0.58
	60	7.68	7.67	7.64	0.95	0.73	0.75	0.56	0.44	0.52
	90	7.86	7.75	7.84	0.53	0.75	0.55	0.34	0.36	0.46

Supplementary Table 2. Effect of application of different concentrations of nano-chitosan-urea composite and conventional urea fertilizer on the percent nitrogen content in plant root, shoot and tuber tissues.

Conc.	Shoot			Root			Tuber			MEAN
	CU	NCUC	CS	CU	NCUC	CS	CU	NCUC	CS	
**0**	0.94±0.01d	0.93±0.00d	0.93±0.00d	0.58±0.01d	0.61±0.02d	0.61±0.02d	1.37±0.01d	1.35±0.01d	1.36±0.01d	**0.96**
**50**	1.01±0.00c	1.76±0.01c	1.40±0.01c	0.81±0.04c	1.85±0.01c	1.34±0.02c	1.51±0.02c	2.22±0.01c	1.79±0.01c	**1.52**
**75**	1.23±0.03b	1.80±0.00b	1.43±0.01b	0.99±0.03b	2.23±0.02b	1.42±0.01b	1.67±0.01b	2.44±0.02b	1.95±0.01b	**1.68**
**100**	1.46±0.03a	1.88±0.02a	1.54±0.00a	1.60±0.00a	2.48±0.01a	1.65±0.02a	1.82±0.02a	2.77±0.04a	2.11±0.03a	**1.92**
**Mean**	**1.16**	**1.59**	**1.32**	**0.99**	**1.79**	**1.25**	**1.59**	**2.19**	**1.8**	

Means within the sub-factor followed by a different letter in a column are significantly different at $p \leq 0.05$ according to a pair-wise comparison of least square means

Supplementary Table 3. Analysis of variance of N-fertilizer source and dose of application (N-level) on vegetative and yield attributes of potato cv. Kufri Pukhraj.

Variables	DF	Fresh shoot wt. (g)	Dry shoot wt. (g)	No. of sprouts	Avg. wt. tuber per plant (g)	Tuber wt. per plant (g)	No. of tubers non-marketable	No. of tubers marketable
Source	2	102.37***	117.61***	0.58ns	6.07ns	3229.86*	5.02**	5.44**
N-level	3	229.70***	226.81***	4.33***	42.56**	25650.00***	4.55**	13.40***
Source* N-level	6	14.56***	15.54***	0.36ns	19.63*	590.97ns	10.87***	2.29*

* $p \leq 0.05$, **= $p \leq 0.01$, and ***=$p \leq 0.001$

Supplementary Table 4. Effect of application of different concentrations of nano-chitosan-urea composite and conventional urea fertilizer on the yield attributes of potato

Conc (%)	Average weight of tubers per pot (g)			Total weight of tubers per pot (g)			Mean
	CU	NCUC	CS	CU	NCUC	CS	
**0**	19.03±0.97d	21.06±0.85d	20.23±1.52c	216.67±22.07d	225.00±14.45c	216.67±22.07d	119.7
**50**	23.08±1.11a	22.14±1.07c	16.33±0.46d	300.00±14.45b	316.67±8.34b	266.67±8.34c	157.5
**75**	22.14±0.60c	25.60±1.14a	23.73±1.51b	325.00±14.45a	375.00±14.45a	348.33±25.90a	186.63
**100**	22.96±0.28b	23.21±1.03b	26.67±1.67a	283.33±10.94c	316.67±8.34b	283.33±8.34b	159.36
**Mean**	21.8	23.01	21.74	281.25	315.83	278.75	

	No. of non-marketable (<35 g) tubers			No. of marketable (>35 g) tubers			
**0**	9.00±0.58b	8.33±0.33c	9.67±0.33c	2.33±0.33c	2.33±0.33d	1.67±0.88d	5.55
**50**	8.67±0.33c	8.67±0.33b	13.00±0.58a	4.33±0.33a	5.67±0.33b	3.33±0.33c	7.27
**75**	10.33±0.33a	8.00±0.00d	11.00±0.58b	4.33±0.33a	6.67±0.33a	3.67±0.33b	7.33
**100**	8.67±0.33c	10.33±0.33a	6.67±0.33d	3.67±0.33b	3.33±0.33c	4.00±0.00a	6.11
**Mean**	9.16	8.83	10.08	3.66	4.50	3.16	

Means within the sub-factor followed by a different letter in a column are significantly different at $p \leq 0.05$ according to a pair-wise comparison of least square means
